# Supplementary material for: Multimodal binding and inhibition of bacterial ribosomes by the antimicrobial peptides Api137 and Api88
Source: Nat Commun. 2024 May 10;15:3945. doi: 10.1038/s41467-024-48027-4 (PMC11087509; doi:10.1038/s41467-024-48027-4)
Supplement: Supplementary file 1 — Supplementary Information [file 41467_2024_48027_MOESM1_ESM.pdf]

SUPPLEMENTARY INFORMATION

Multimodal binding and inhibition of bacterial ribosomes by the antimicrobial peptides Api137 and Api88

Simon Lauer[1,2,#], Maren Reepmeyer[3,4,#], Ole Berendes[5], Dorota Klepacki[6], Jakob Gasse[3,4], Sara Gabrielli[5], Helmut Grubmüller[5], Lars V. Bock[5], Andor Krizsan[3,4], Rainer Nikolay[1,7] ✉, Christian M. T. Spahn[1] ✉ and Ralf Hoffmann[3,4] ✉

[#] These authors contributed equally.

[1]Institute of Medical Physics and Biophysics, Charité – Berlin University of medicine, corporate member of Freie Universität Berlin and Humboldt Universität zu Berlin, Berlin, Germany.

[2]Humboldt-Universität zu Berlin, Institut für Biologie, 10099 Berlin, Germany.

[3]Institute of Bioanalytical Chemistry, Faculty of Chemistry and Mineralogy, Universität Leipzig, Leipzig, Germany.

[4]Center for Biotechnology and Biomedicine, Universität Leipzig, Leipzig, Germany.

[5]Theoretical and Computational Biophysics Department, Max Planck Institute for Multidisciplinary Sciences, Göttingen, Germany.

[6]Department of Pharmaceutical Sciences, University of Illinois at Chicago, Chicago, IL 60607, USA.

[7]Max Planck Institute for Molecular Genetics, Department of Genome Regulation, Ihnestrasse 63-73, 14195, Berlin, Germany.

✉ Corresponding authors: Dr. Rainer Nikolay (nikolay@molgen.mpg.de), Prof. Christian Spahn (christian.spahn@charite.de) and Prof. Ralf Hoffmann (bioanaly@rz.uni-leipzig.de)

Table of contents

Supplementary Fig. 1: Purification of 70S ribosomes S3
Supplementary Fig. 2: Ribosome binding by PrAMPs and other antibiotics S4
Supplementary Fig. 3: Characterization of Api88(Y7B)-cross-linked 70S ribosomes S5
Supplementary Fig. 4: Tandem mass spectra of peptides used to identify enriched proteins and extracted ion chromatograms (XICs) to assess changes in protein quantities..........S6
Supplementary Fig. 5: Tandem mass spectra of peptides used to identify enriched proteins and corresponding XICs of unspecifically enriched proteins..S8
Supplementary Fig. 6: Sorting schemes, final maps, global and local resolution S9
Supplementary Fig. 7: Cryo-EM structures of 50S•Api137 and 50S•Api88 ....S11
Supplementary Fig. 8: Focused classification of Api88 conformations in the PET S12
Supplementary Fig. 9: Comparison of known PrAMP conformations in the PTC S13
Supplementary Fig. 10: Correlation coefficient between the weight-optimized linear combination of MD ensemble maps and the cryo-EM map S14
Supplementary Fig. 11: Interaction sites of 50S•Api137 and 50S•Api88............................S15
Supplementary Fig. 12: Comparison of C-terminal sites of 70S•Api137, 50S•Api137, and 50S•Api88 complexes S17
Supplementary Fig. 13: Additional binding site of Api88 at the PET exit............................S18
Supplementary Fig. 14: Overall peptide geometry at different binding sites......................S20
Supplementary Fig. 15: Mass spectra and chromatograms obtained for the purified synthetic peptides Api88, Api137, and Onc112 ....S21
Supplementary Fig. 16: Mass spectra and chromatograms obtained for the purified synthetic peptides labelled with 5(6)-carboxyfluorescein (Cf) or biotin (Bio) S22
Supplementary table 1: Molecular dynamics checklist..S23

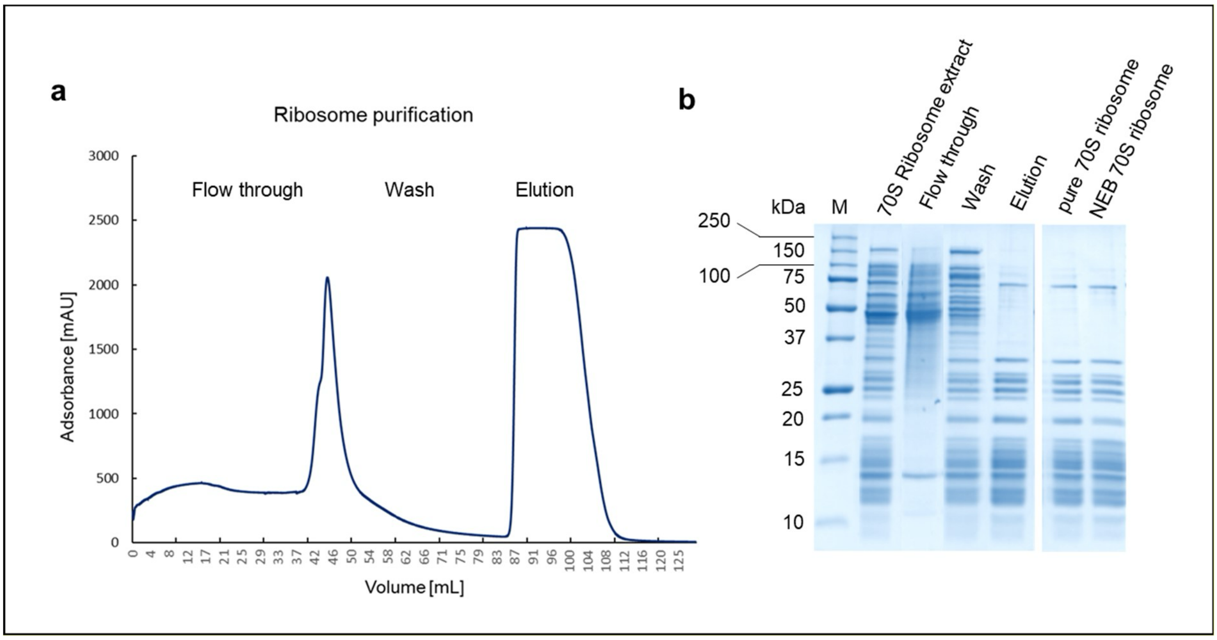

Supplementary Fig. 1: Purification of 70S ribosomes using anion exchange chromatography (AEC). a Chromatogram showing the purification of a 70S ribosomal extract by anion exchange chromatography on a CIM®QA column. Absorbance was measured at 260 nm. The 70S ribosomal extract was loaded in 40% eluent B (20 mmol/L HEPES-KOH, 6 mmol/L $MgCl_2$, 1 mol/L NH_4Cl, pH 7.6, 4 °C) using a flow rate of 2 mL/min, washed with five column volumes, and eluted by a linear gradient of 20% eluent B/min. Collected fractions, indicated by dashed lines, were combined, concentrated, rebuffered in ribosome preparation buffer (20 mmol/L HEPES-KOH, 6 mmol/L $MgCl_2$, 30 mmol/L NH_4Cl, 4 mmol/L β-mercaptoethanol, pH 7.6, 4 °C),and stored at -80°C. **b** Coomassie Brilliant Blue-stained gel of an SDS-PAGE (T=16%) obtained by analyzing the 70S purification steps. First, ribosomal extract containing other bacterial proteins, which was used for further purification by anion exchange chromatography and the received fractions obtained by AEC (panel A), divided into flow through, wash, showing both separated bacterial proteins from 70S ribosome, the elution of 70S without other proteins, the concentrated pure *E. coli* 70S ribosome obtained by AEC and used for binding and competitive assays in the current study, and an *E. coli* 70S ribosome obtained from New England Biolabs (*E. coli* Ribosome, P0763S, New England Biolabs, Massachusetts, US) as a reference, which was used in the *in vitro* transcription-translation (iTT) assay. Source data are provided as a Source Data file.

a

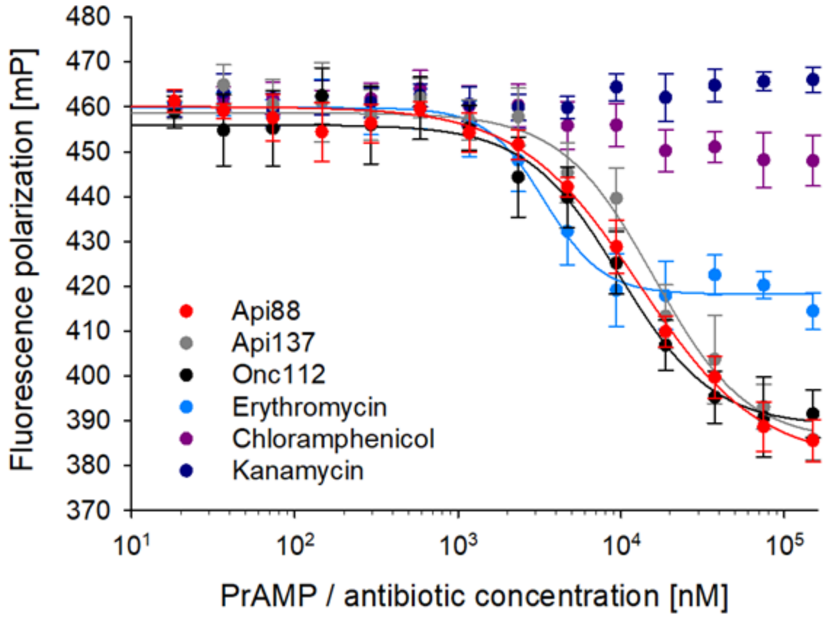

b

	Inhibition of Cf-Api88 binding	
**PrAMP/Antibiotic**	**IC_{50} [µmol/L]**	**K_i [µmol/L]**
Api88	12.8 ± 1.3	2.45 ± 0.49
Api137	15.9 ± 2.3	3.03 ± 0.88
Onc112	9.8 ± 1.3	1.87 ± 0.49
Erythromycin (50S)	3.4 ± 0.3	0.65 ± 0.12
Chloramphenicol (50S)	>100	n.c.
Kanamycin (30S)	>100	n.c.

Supplementary Fig. 2: Ribosome binding by PrAMPs and other antibiotics. a Fluorescence polarization assay of purified 70S ribosome and Cf-Api88 in competition with Api88 (red), Api137 (gray), Onc112 (black), erythromycin (light blue) and chloramphenicol (purple), which both bind to the 50S subunit, and kanamycin (dark blue), which binds to the 30S subunit. Error bars show the deviation from the mean of duplicates in two independent experiments (n=4). **b** IC_{50} and K_i values calculated from the binding curves shown in **a**. n.c.= not calculable.

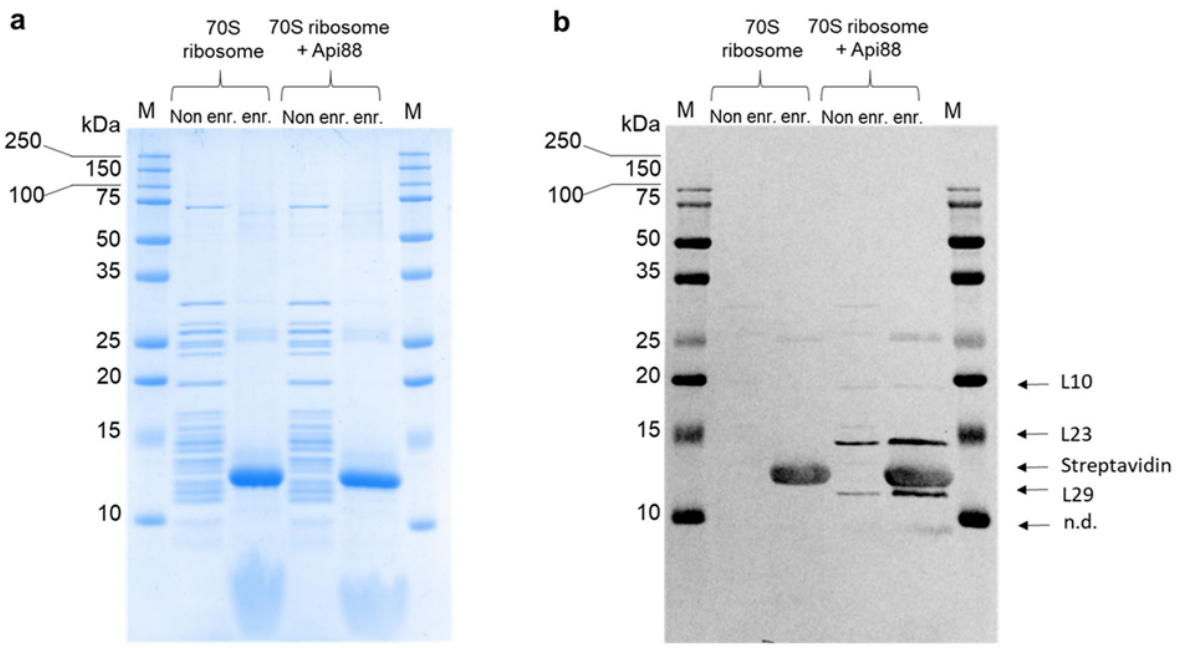

c

MW of signal in western blot	Protein	Sequence coverage	Biologcal replicate	Peak area in 70S ribosome	Peak area in 70S ribosome + Api88	Ratio
~20 kDa	L10	35.8 %	1	809929	3615172	4.46
			2	471236	2816688	5.98
~ 15 kDa	L23	32 %	1	25976	619541	23.85
			2	n.d.	718370	n.d.
~ 12 kDa	L29	22.2 %	1	n.d.	75857	n.d.
			2	n.d.	18288	n.d.

Supplementary Fig. 3: SDS-PAGE and Western blot of purified 70S ribosomes UV-irradiated in the absence or presence of biotin-SG-Api88(Y7B). Visualization of irradiated (load) and affinity-enriched 70S ribosome samples by **a** SDS-PAGE (T = 16%) and **b** Western blot. The shown gel and blot represent one of two replicates. **c** Proteins represented by the four bands detected at higher intensities were identified as ribosomal 50S proteins L10, L23, and L29 after tryptic in-gel digestion by nanoUPLC-ESI-QTOF-MS. Shown are the peak areas of all identified peptides representing the corresponding protein in the absence or presence of Bio-SG-Api88(Y7B) and the peak area ratio. n.d. = not defined. Source data are provided as a Source Data file.

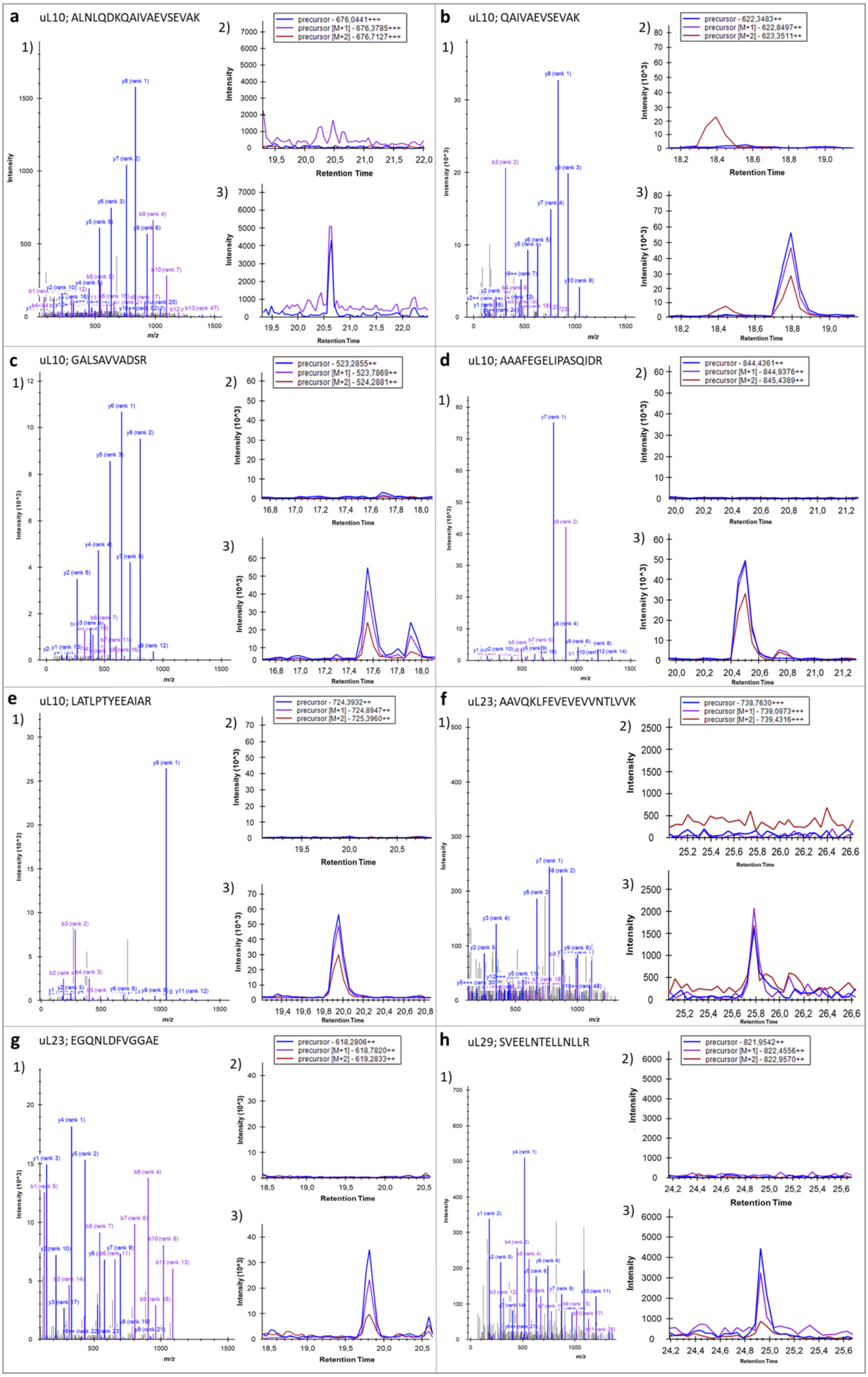
a
uL10; ALNLQDKQAIVAEVSEVAK
precursor - 676,0441+++
precursor [M+1] - 676,3785+++
precursor [M+2] - 676,7127+++
Retention Time
Intensity
m/z
b
uL10; QAIVAEVSEVAK
precursor - 622,3483++
precursor [M+1] - 622,8497++
precursor [M+2] - 623,3511++
Intensity (10^3)
c
uL10; GALSAVVADSR
precursor - 523,2855++
precursor [M+1] - 523,7869++
precursor [M+2] - 524,2881++
d
uL10; AAAFEGELIPASQIDR
precursor - 844,4361++
precursor [M+1] - 844,9376++
precursor [M+2] - 845,4389++
e
uL10; LATLPTYEEAIAR
precursor - 724,3932++
precursor [M+1] - 724,8947++
precursor [M+2] - 725,3960++
f
uL23; AAVQKLFEVEVEVVNTLVVK
precursor - 738,7630+++
precursor [M+1] - 739,0973+++
precursor [M+2] - 739,4316+++
g
uL23; EGQNLDFVGGAE
precursor - 618,2806++
precursor [M+1] - 618,7820++
precursor [M+2] - 619,2833++
h
uL29; SVEELNTELLNLLR
precursor - 821,9542++
precursor [M+1] - 822,4556++
precursor [M+2] - 822,9570++

Supplementary Fig. 4: Tandem mass spectra of peptides used to identify enriched proteins and extracted ion chromatograms (XICs) to assess changes in protein quantities. Ribosomal proteins uL10, uL23 and uL29 were identified based on eight peptides **(a-h)** identified by nanoUPLC-ESI-QTOF-MS of 70S ribosomes cross-linked in the absence (control) or presence of biotin-SG-Api88(Y7B) after streptavidin enrichment, SDS-PAGE, and in-gel digestion with trypsin. Each panel (**a-h**) shows the peptide sequence deduced from the b- and y-series of the tandem mass spectrum (**1**). XICs of the corresponding precursor ion masses of the indicated charge states (++ and +++ denote charge states of 2 and 3) for the monoisotopic (blue) and two higher isotopic signals (purple and red for +1 and +2, respectively) obtained for the control (**2**) and biotin-SG-Api88 (Y7B) (**3**) samples. The unenriched/enriched ratios corresponding to the total proteins are shown in Supplementary Fig. 3 c.

a

Accession	Description	Coverage (%)	Unique Peptide
P22629	Streptavidin	73	75
P0A7V3	30S ribosomal protein S3	74	35
P60422	50S ribosomal protein L2	63	32
P0AG67	30S ribosomal protein S1	43	24
P06959	Dihydrolipoyllysine-residue acetyltransferase component of pyruvate dehydrogenase complex	40	21
P0A853	Tryptophanase	43	21
P0AFG8	Pyruvate dehydrogenase E1 component	25	20
P02359	30S ribosomal protein S7	68	19
P0A7V0	30S ribosomal protein S2	68	18
P0A7V8	30S ribosomal protein S4	47	14
P0AA10	50S ribosomal protein L13	64	12
P61175	50S ribosomal protein L22	64	11
P0A9P0	Dihydrolipoyl dehydrogenase	26	10

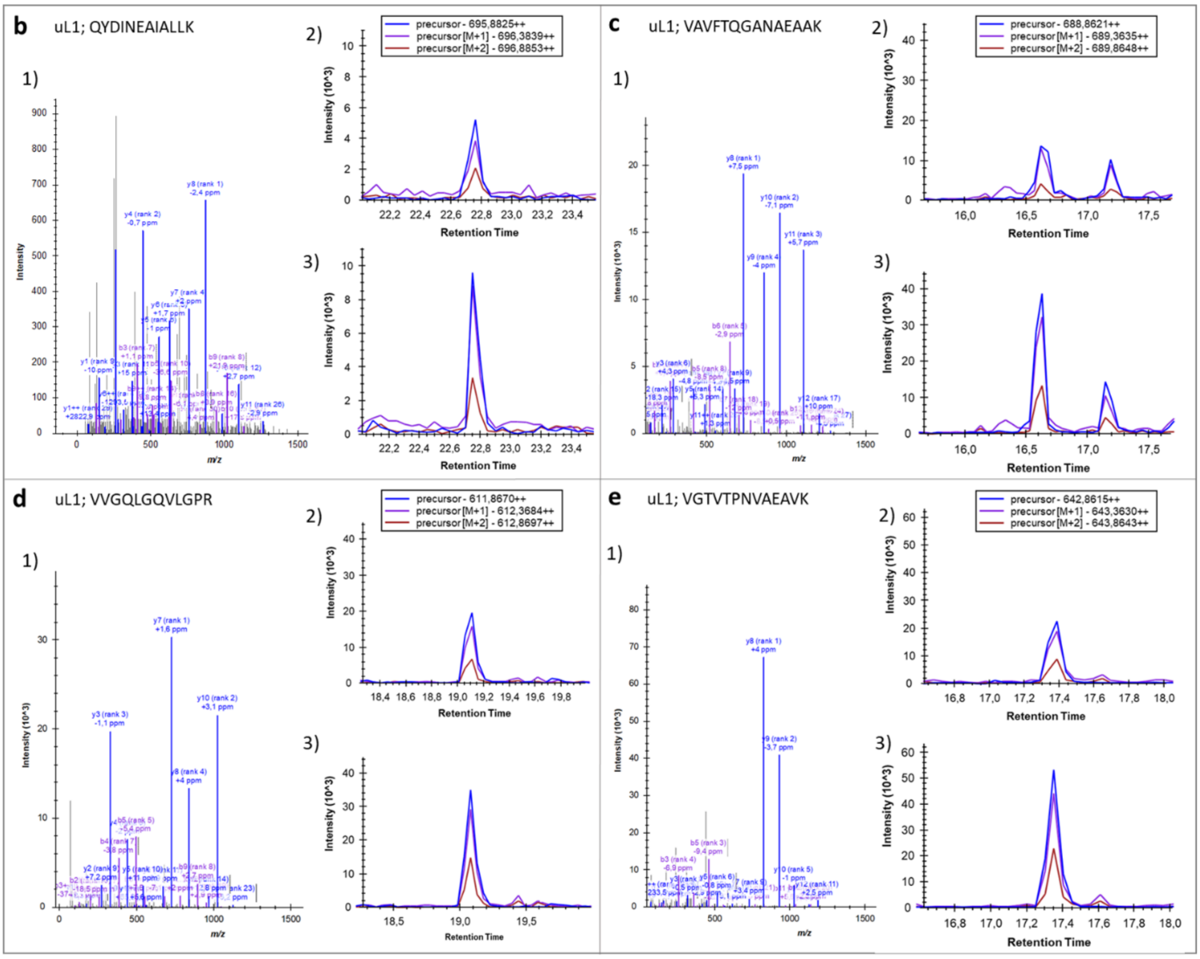

Supplementary Fig. 5: Tandem mass spectra of peptides used to identify enriched proteins and corresponding XICs of unspecifically enriched proteins. In addition to the enriched proteins described in Supplementary Fig. 4, many peptides from different proteins were detected with similar intensities in the XICs of the control and biotin-SG-Api88(Y7B) samples (**a**), as exemplified for the ribosomal protein uL1 **(b-e)**. Each panel shows the tandem mass spectra (1) and the XICs of the control (2) and biotin-SG-Api88(Y7B) (3) samples. Further details are provided in the legend of Supplementary Fig. 4.

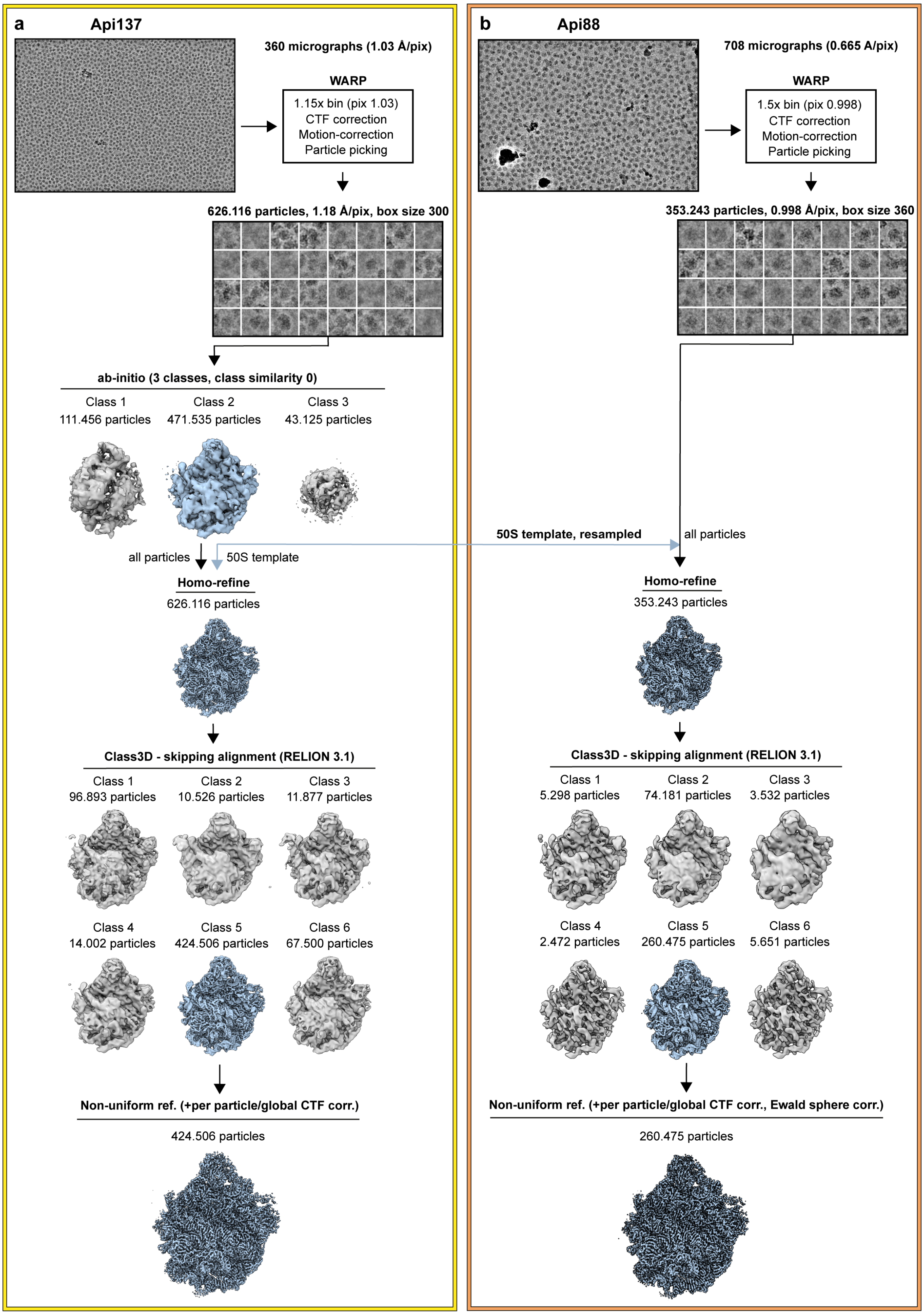

Supplementary Fig. 6: Sorting schemes for the 50S•Api137 and 50S•Api88 data sets. Data shown for **a** 50S•Api137 and **b** 50S•Api88 complexes. Representative micrographs for each dataset are shown, low-pass filtered to 5 Å resolution. Pre-processing steps were performed in WARP. 50S•Api137

particles were extracted using a box size of 300 at a pixel size of 1.18. 50S•Api88 particles were extracted using a box size of 360 at a pixel size of 0.998. An internal 50S template was generated using 50S•Api137 particles and a multi-class ab initio reconstruction. Subsequently, all particles from both datasets were aligned to the consensus map, followed by 3D classification skipping alignment in RELION 3.1. Highly resolved classes from both datasets were refined using non-uniform refinement with CTF correction in cryoSPARC 3.1. For 50S•Api88, a final Ewald sphere correction was performed.

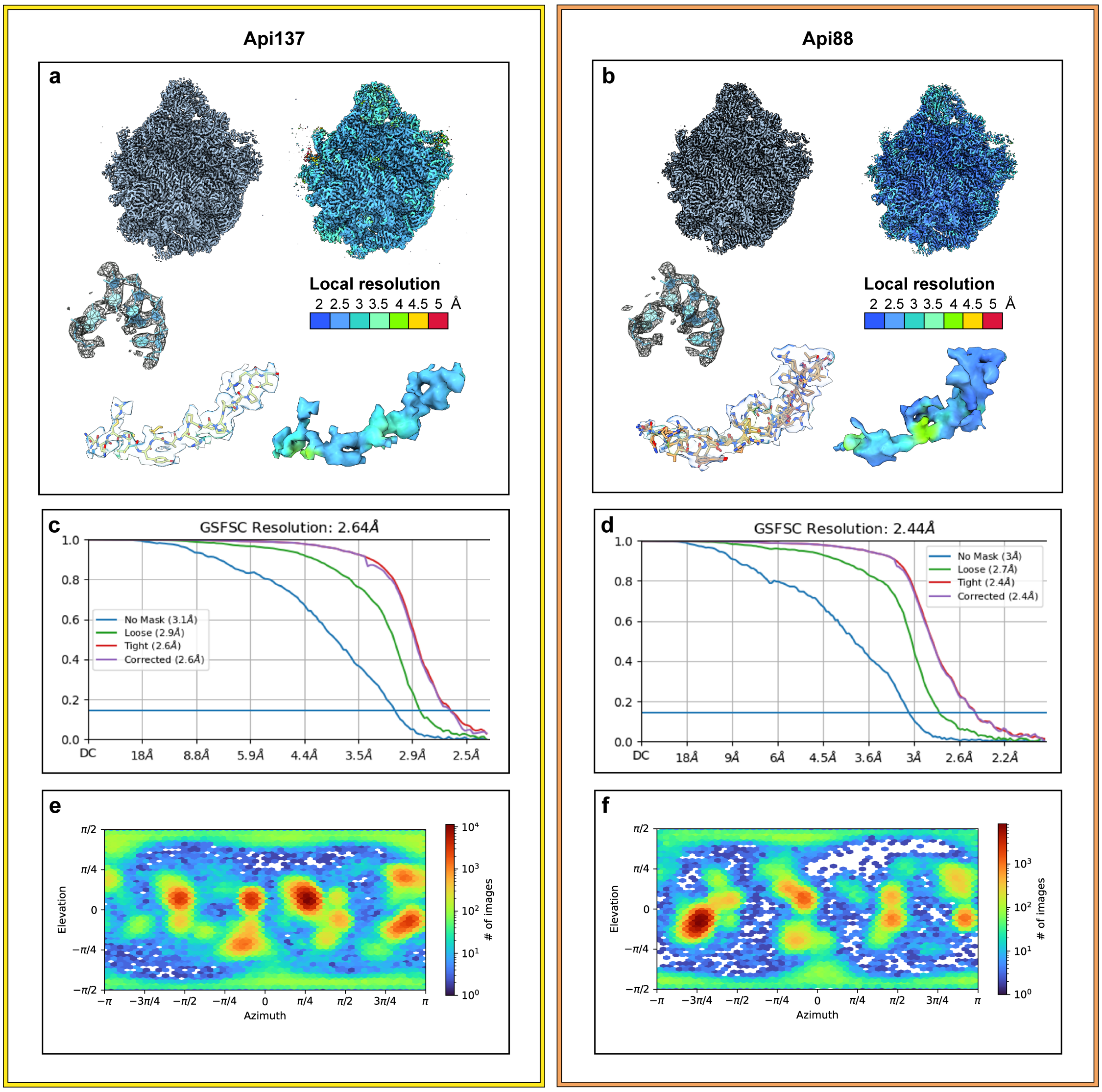

Supplementary Fig. 6: Validation of final 50S•Api137 and 50S•Api88 maps. a, **b** Sharpened maps, local resolution maps (resolution range: 2-5 Å), and local resolution of apidaecins at the PET binding site of final maps are shown for **a** 50S•Api137 and **b** 50S•Api88. Local maps are shown for randomly selected rRNA helices within the subunit's core as representative of map quality. **c**, **d** Global FSC curves for 50S•Api137 (**c**) and 50S•Api88 maps (**d**). **e**, **f** Angular distribution plots of final reconstructions for 50S•Api137 (**e**) and 50S•Api88 (**f**).

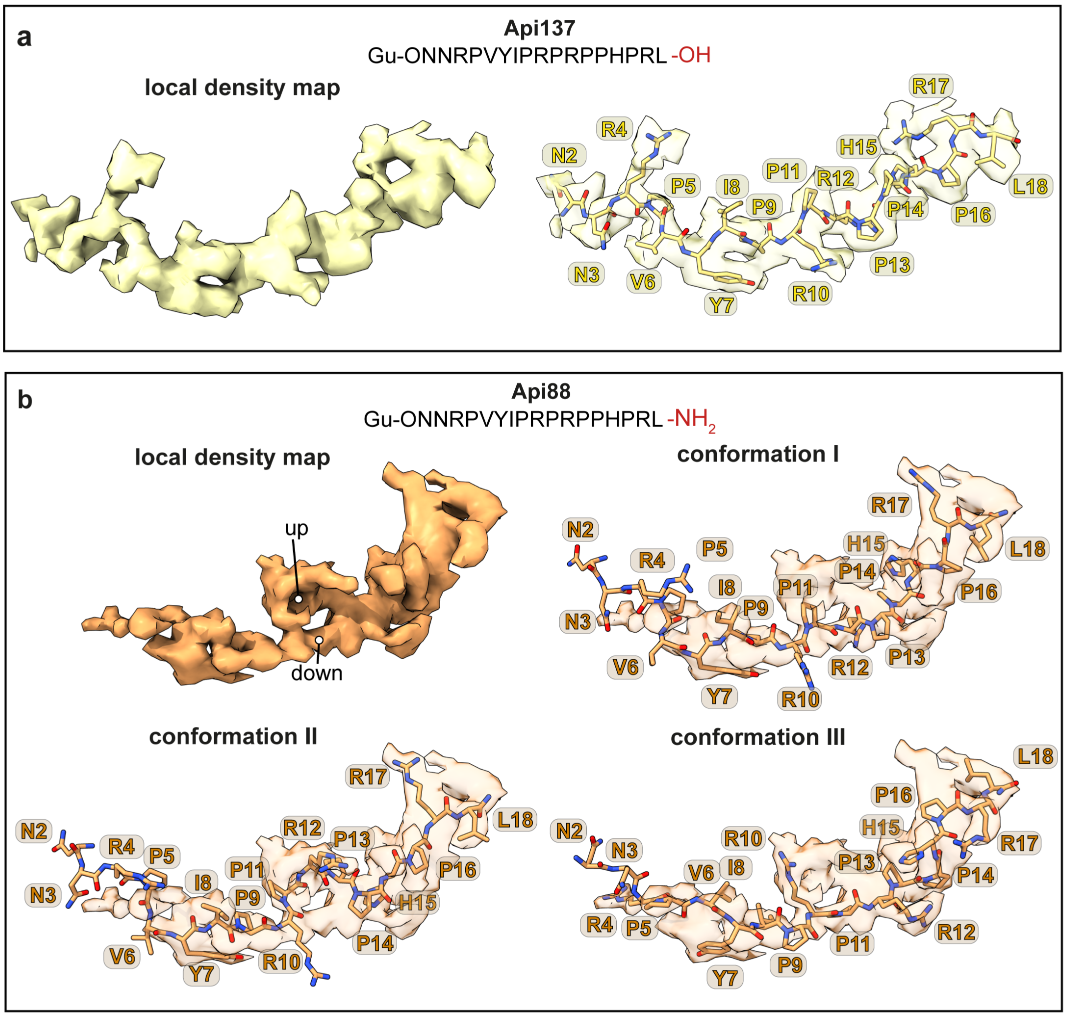

Supplementary Fig. 7: Cryo-EM structures of 50S•Api137 and 50S•Api88. a Left: local cryo-EM map of Api137 (yellow). Right: Api137 residues modeled in the cryo-EM density (transparent yellow surface). **b** Local cryo-EM map of Api88 (orange) and the putative atomic models fitting conformations I-III (transparent orange surfaces).

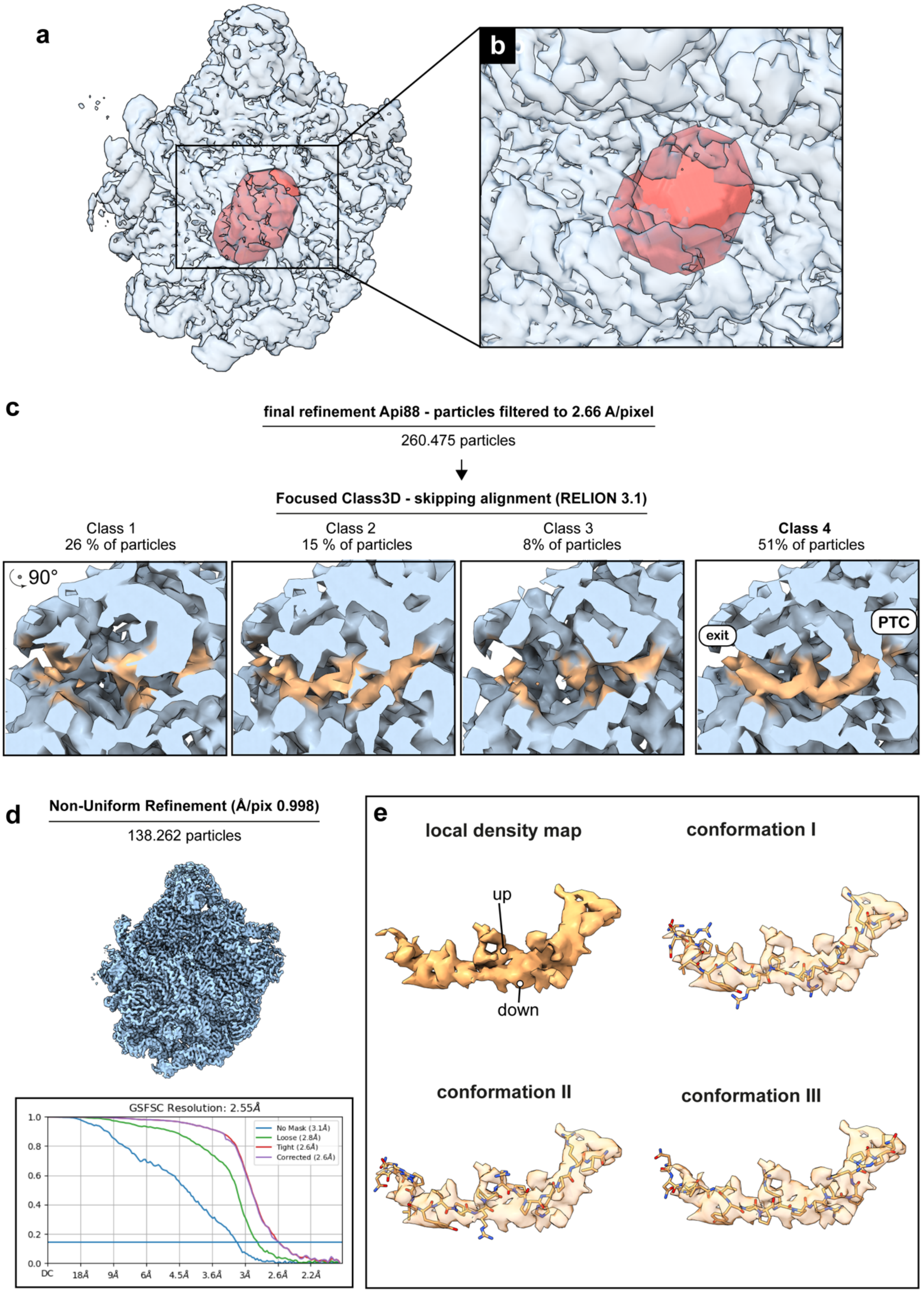

Supplementary Fig. 8: Focused classification of Api88 conformations in the PET. a and b A binary mask (red) was generated for the PET binding site **c** Final particle subset of 50S•Api88 was filtered to 2.66 Å/pixel and subjected to a focused classification in Relion 3.1. Class 4 showed a defined Api88 density within the PET. **d** Selected particles were subjected to a Non-Uniform Refinement. **e** Local density map was more pronounced, but still showed features of all modeled conformations.

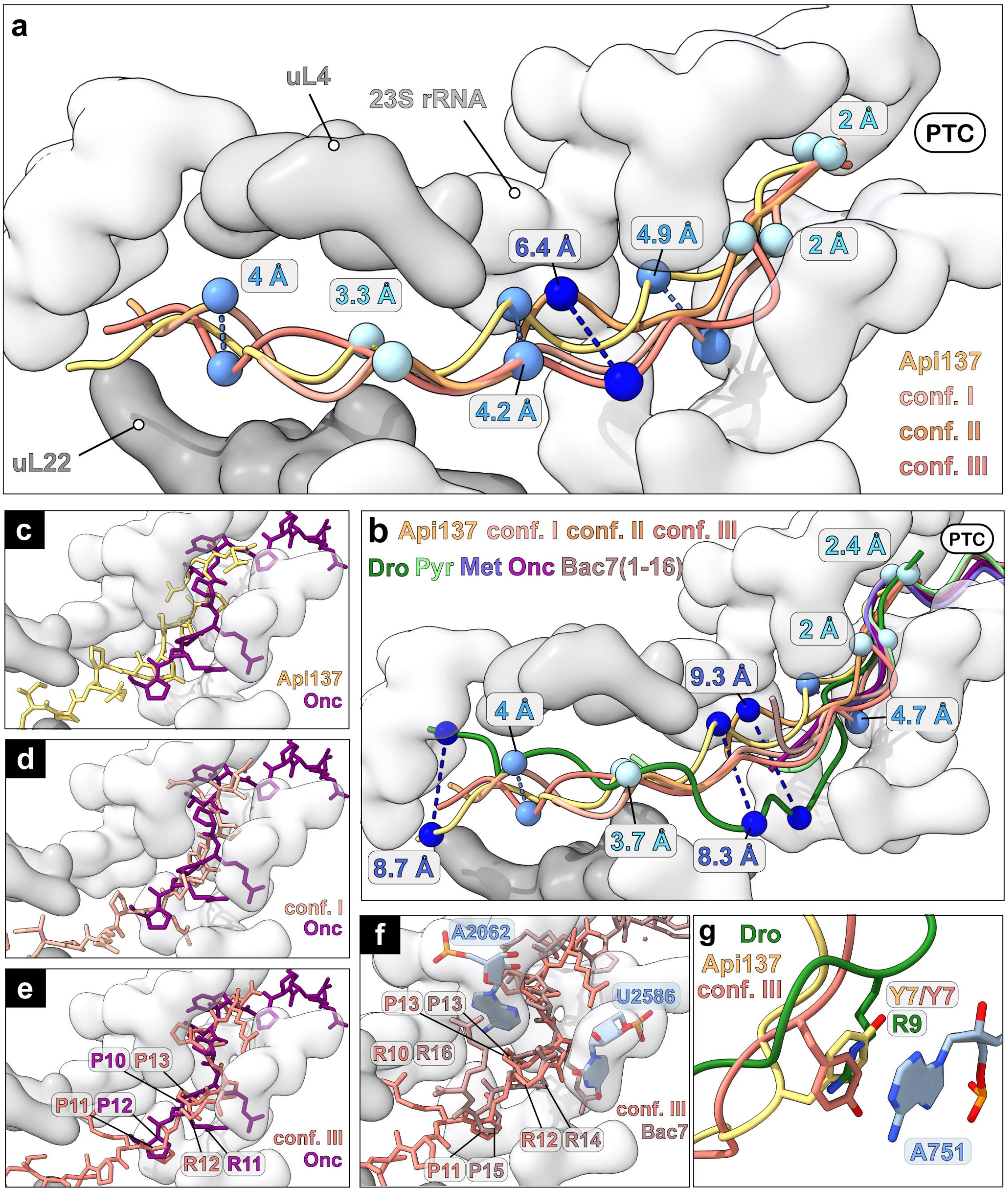

Supplementary Fig. 9: Comparison of known PrAMP conformations in the PTC including Api137 and Api88. a Conformational space occupied by conformations I (pale orange), II (orange), III (light red) of Api88 and Api137 (yellow) within the tunnel binding site. Distances between selected C_α atoms are indicated using blue dots and dashed lines. Distances are color-coded (<4 Å = light blue, 4-6 Å cornflower blue, >6 Å dark blue. **b** Conformational space occupied by Api88, Api137, and other PrAMPs within the tunnel (Onc112 (PDB: 4ZER[9,10], Met (PDB: 5FDU[27,28], Pyr (PDB: 5FDV[27,28], Bac7(1-16) (PDB: 5F8K[27,28], Dro (PDB: 8ANA[29]. Distances between selected C_α atoms are indicated using blue dots and dashed lines. Distances are color-coded (<4 Å = light blue, 4-6 Å cornflower blue, >6 Å dark blue. **c-e** Comparison of **c** Api137, **d** Api88 conformation I, and **e** Api88 conformation III with the position and conformation of Onc112). f, Comparison of Api88 conformation III with the position and conformation

of Bac7(1-16; brown). **g** Residues of Api137(Y7), Api88(Y7), and Dro(R9) interacting with A751 of the 23S rRNA.

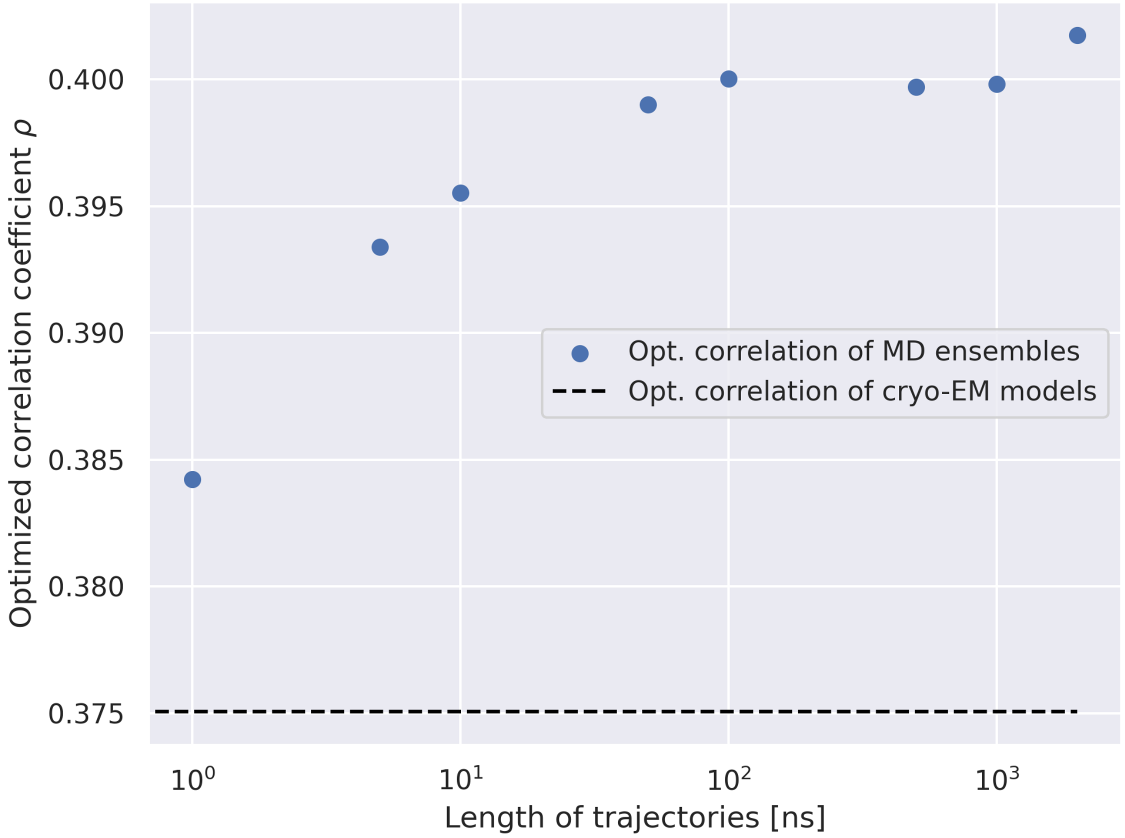

Supplementary Fig. 10: Correlation coefficient between the weight-optimized linear combination of MD ensemble maps and the cryo-EM map. Each data point includes frames from 5 independent trajectories up to the specified time for the calculation of the maps (starting from the 70 ns equilibration period). The dashed black line shows the correlation coefficient for the weight-optimized initial cryo-EM models.

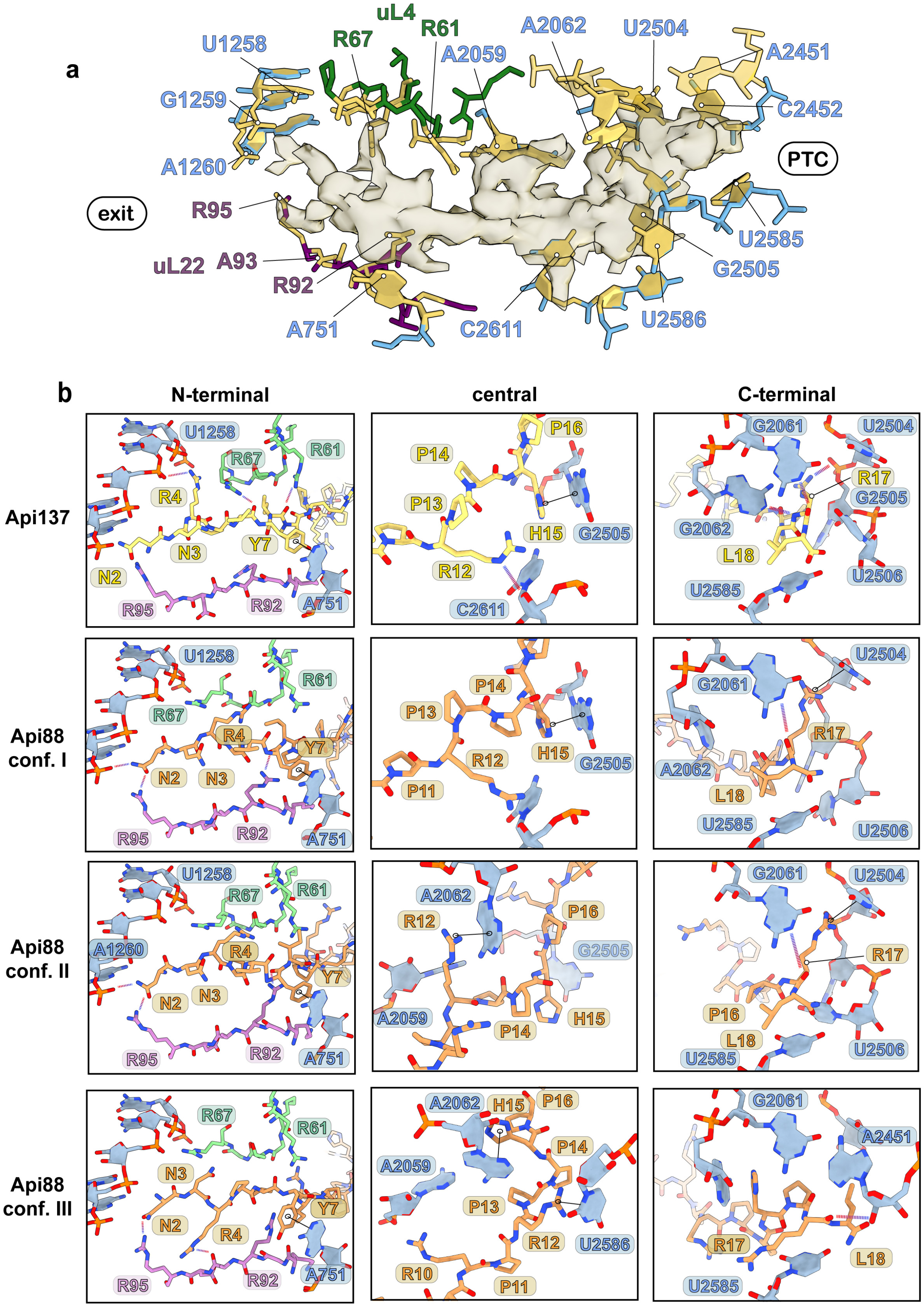

Supplementary Fig. 11: Interaction sites of 50S•Api137 and putative 50S•Api88 conformations. a Tunnel environment of Api88. Residues of the 50S subunit and Api88 interacting within a distance of

≤4 Å are highlighted in yellow. The density map indicates multiple conformations. **b** Major interaction sites of Api137 and the three putative Api88 conformations with the large subunit, in the N-terminal (left), central (middle), and C-terminal (right) regions. Hydrogen bonds are shown as dashed lines, stacking interactions as connected black circles.

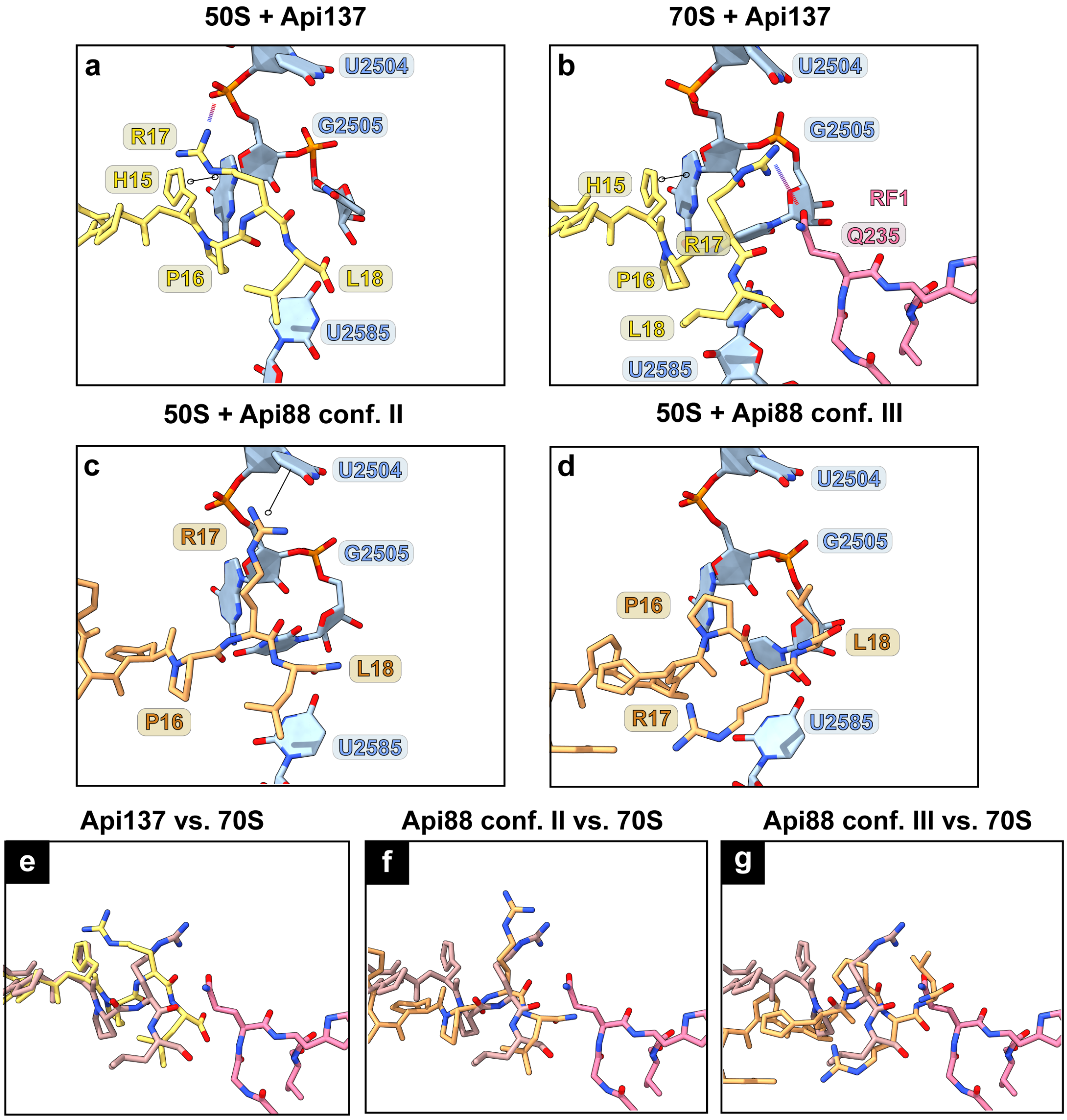

Supplementary Fig. 12: Comparison of C-terminal sites of 70S•Api137, 50S•Api137, and 50S•Api88 complexes. Modeled residues from **a** 50S•Api137, **b**, 70S•Api137, **c** 50S•Api88 conformation I, and **d** 50S•Api88 conformation III complexes. Positions of the Api137 and Api88 residues P16, R17, and L18 (50S•Api137: yellow, 50S•Api88: orange, 70S•Api137: salmon) and 23S rRNA residues G2505 and U2585 (blue) are shown. Release factor 1 (RF1, purple) residue Q235 is shown in the 70S•Api137 complex. **e**-**g** Superimposition of apidaecin models from this study and reported for the Api137-RF1 complex (PDB: 5O2R[[11]]). Hydrogen bonds are shown as dashed lines, stacking interactions as connected black circles.

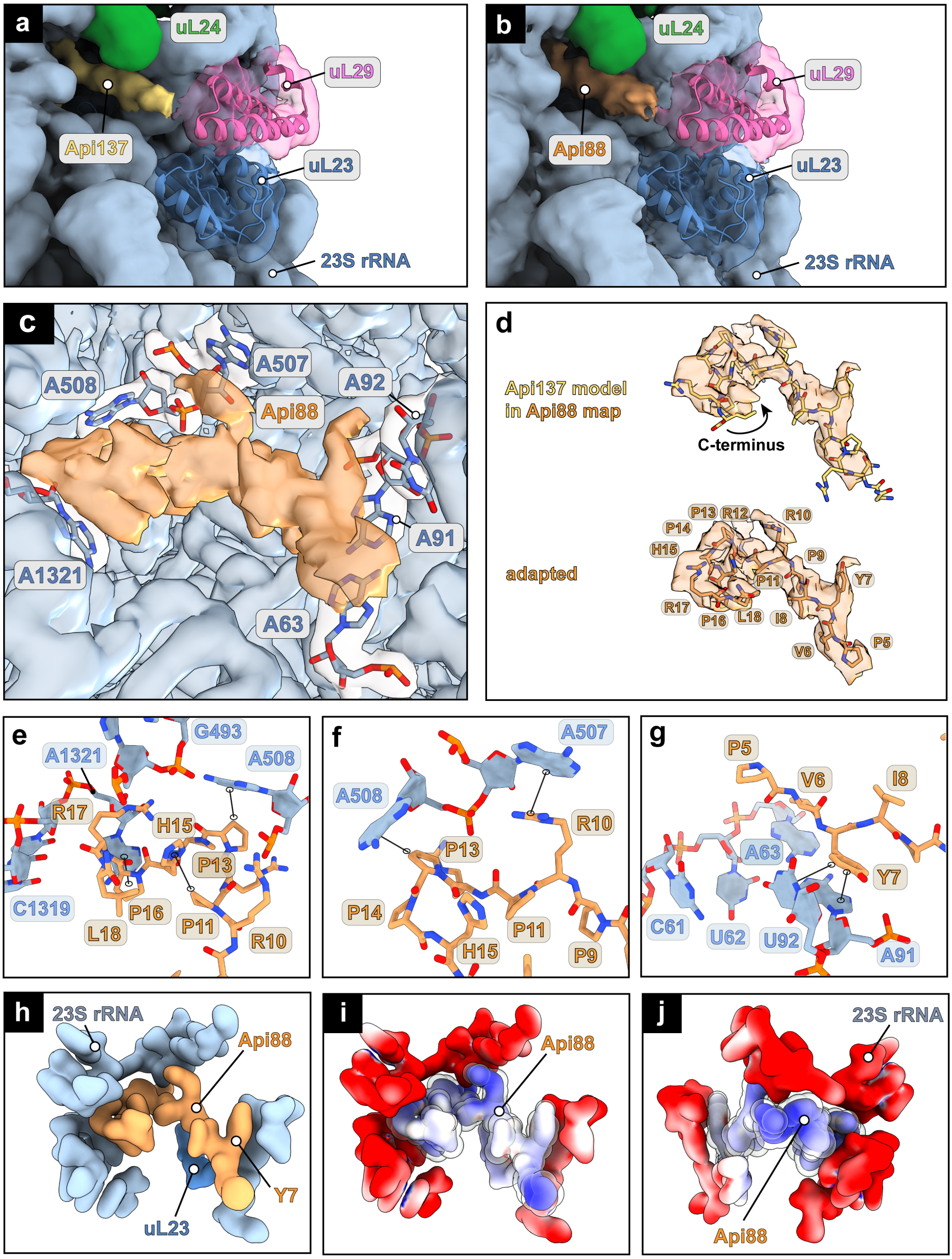

Supplementary Fig. 13: Additional binding site of Api88 at the PET exit. N-terminal regions of **a** Api137 and **b** Api88 at the PET exit. Cryo-EM maps of 50S•Api137 and 50S•Api88 were low-pass filtered and colored. Atomic models of uL23 and uL29 are shown in transparent local maps. **c** Location and assigned Api88 density in orange illustrating rRNA interacting sites with the 23S rRNA. **d** Top: Modeled Api137 conformation at the tunnel exit binding site fit into the local Api88 density map. Arrow indicates

conformational difference of the C-terminus. Bottom: adapted Api88 model in the local Api88 density map. **e-g** More detailed interactions. Hydrogen bonds are shown as dashed lines, stacking interactions as connected black circles. **h** Surface model of Api88 and surrounding rRNA at 4Å resolution. **i** Electrostatic surface potential of Api88 and surrounding rRNA sites. Surface charges are shown in red (-10 kcal/(mol·*e*), white (0 kcal/(mol·*e*) and blue (10 kcal/(mol·*e*). j, rotated view of **I**. For clarity, Api137 and Api88 are highlighted using a transparent sphere model in electrostatic representations.

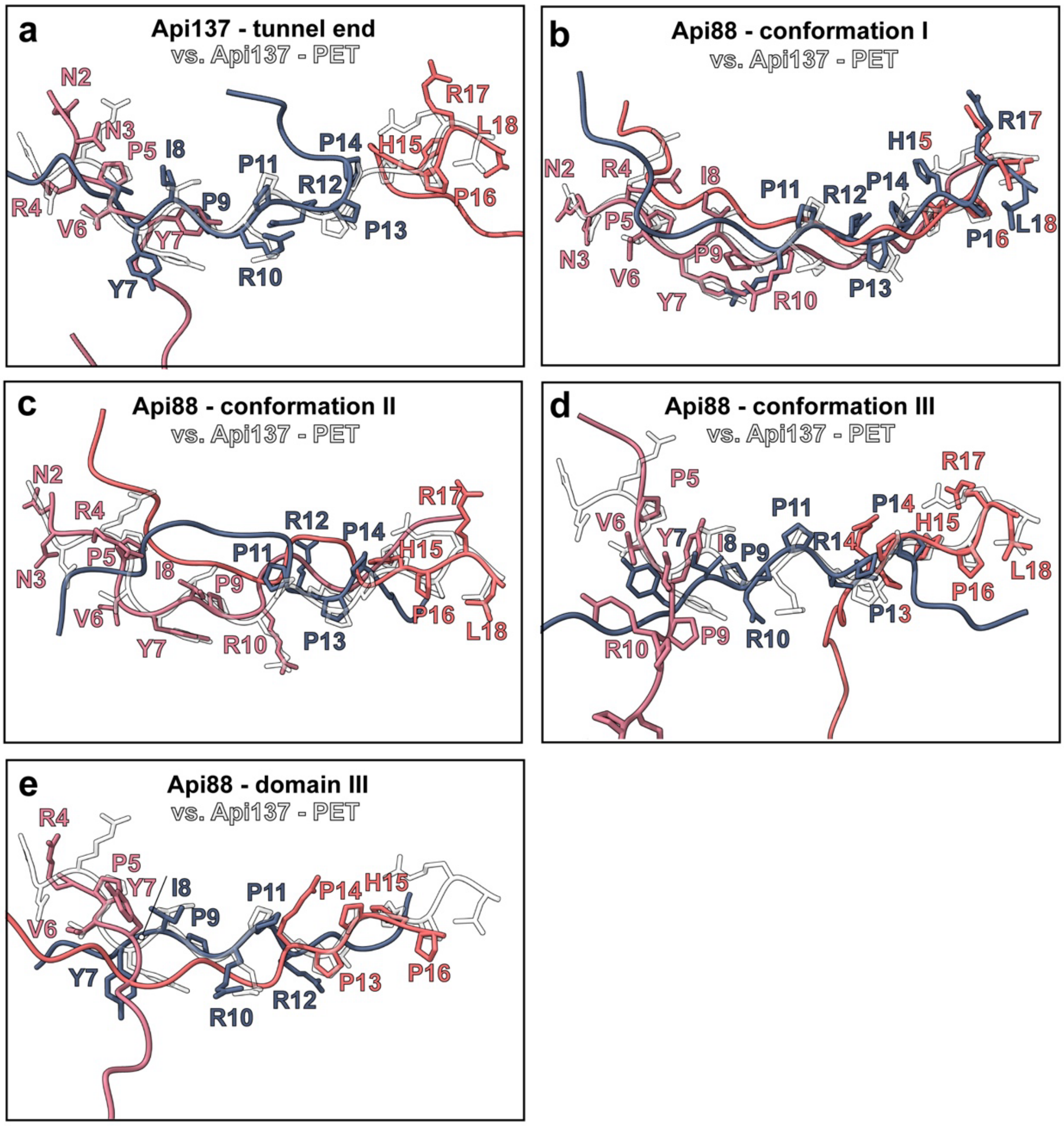

Supplementary Fig. 14: Overall peptide geometry at different binding sites. Canonical conformation of Api137 within the PET is shown in grey for comparison. N-terminal (dark red), central (blue) and C-terminal (light red) sections of modeled residues at different sites were rigid body docked into a 4 Å molmap of Api137. Residues used for docking are labeled using respective color and are shown as sticks **a** Modeled residues of Api137 at the tunnel end. **b** Modeled residues of Api88 conformation I within the PET. **c** Modeled residues of Api88 conformation II within the PET. **d** Modeled residues of Api88 conformation III within the PET. **e** Modeled residues of Api88 at the third binding site.

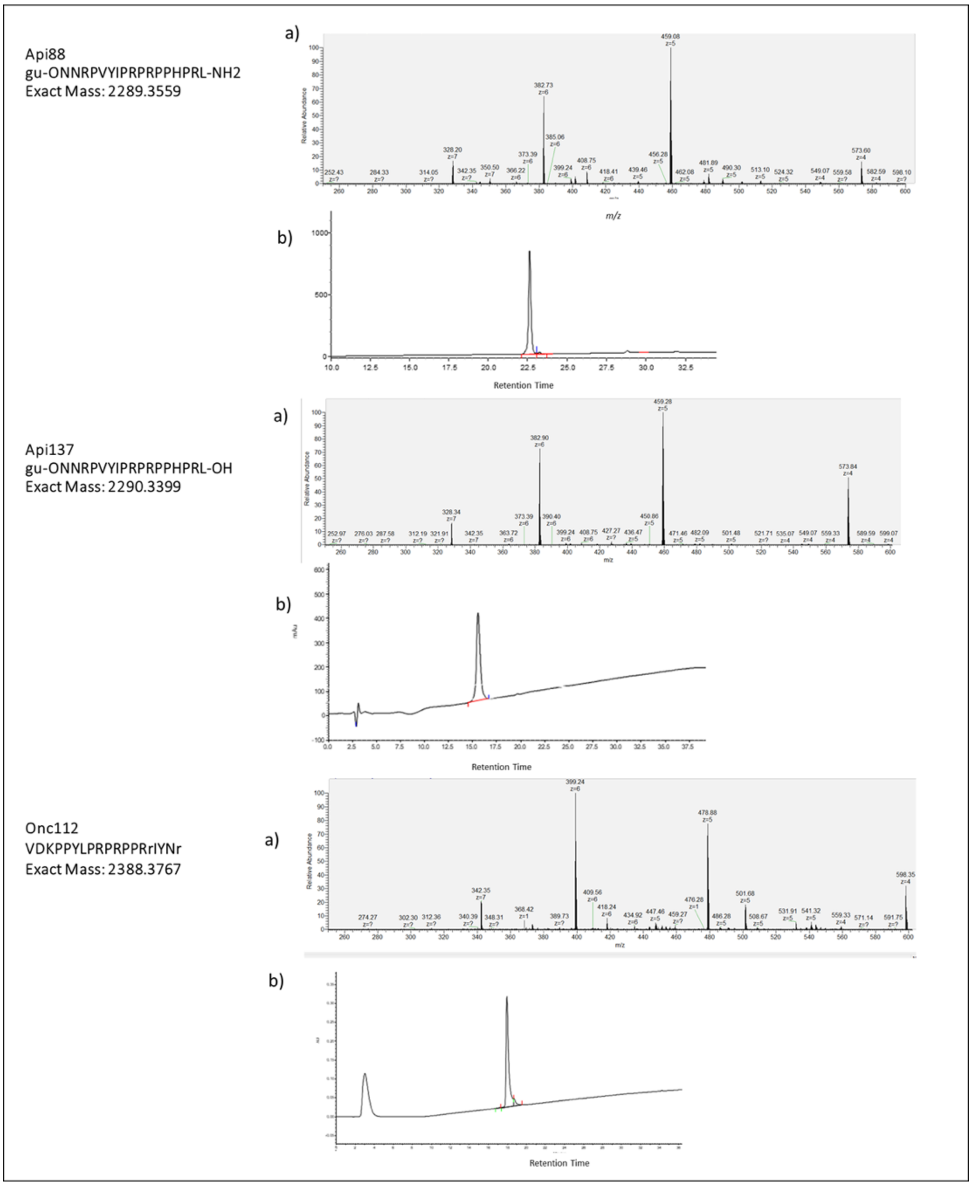

Supplementary Fig. 15: Mass spectra and chromatograms obtained for the purified synthetic peptides Api88, Api137, and Onc112. Left: Peptide names, sequences, and theoretical monoisotopic masses. Right: Mass spectra recorded by ESI-MS **(a)** and reversed-phase chromatograms recording the absorbance at 214nm **(b).**

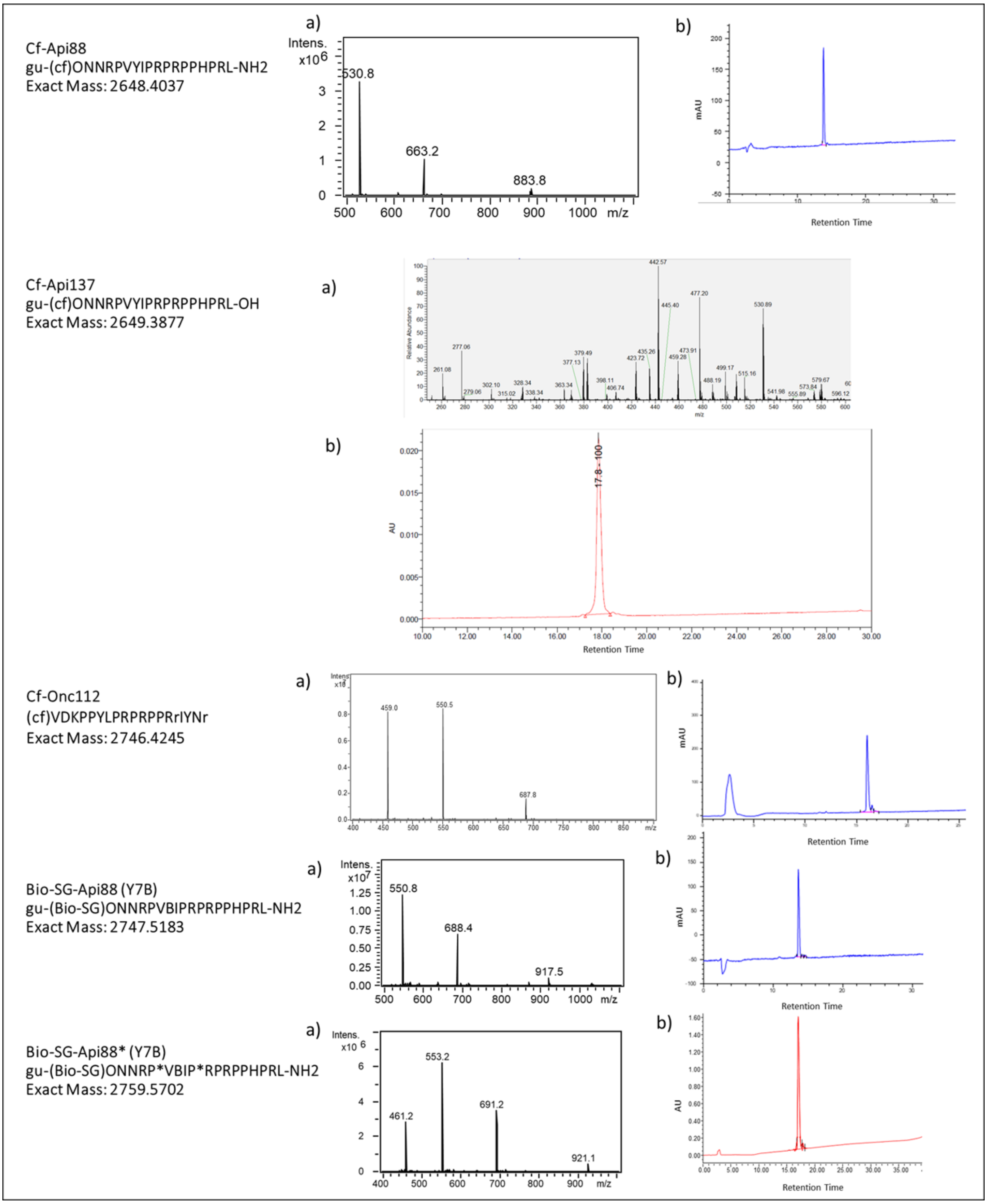

Supplementary Fig. 16: Mass spectra and chromatograms obtained for the purified synthetic peptides labelled with 5(6)-carboxyfluorescein (Cf) or biotin (Bio). Left: Peptide names, sequences, and theoretical monoisotopic masses. Right: Mass spectra recorded by ESI-MS **(a)** and reversed-phase chromatograms recording the absorbance at 214nm **(b).**

Supplementary Tab. 1: MD simulation checklist

**Reliability and reproducibility checklist for molecular dynamics simulations** ***All boxes must be marked YES by acceptance unless an N/A option is available**	**Yes**	**N/A**	**Response (Please state where this information can be found in the text)**
**1. Convergence of simulations and analysis**			
1a. Is an evaluation presented in the text to show that the property being measured has equilibrated in the simulations (*e.g.* time-course analysis)?	☒		Supplementary Figure 10 shows the convergence of the correlation between maps obtained from MD simulations and the cryo-EM map.
1b. Then, is it described in the text how simulations are split into equilibration and production runs and how much data were analyzed from production runs?	☒		Methods: Molecular dynamics (MD) simulations.
1c. Are there at least 3 simulations per simulation condition with statistical analysis?	☒		Five replica per starting conformation of Api88 → Methods: Molecular dynamics (MD) simulations.
1d. Is evidence provided in the text that the simulation results presented are independent of initial configuration?	☒		Evidence is provided in Fig. 3a that the energetically favored region in the conformational space sampled in the simulations deviates largely from the initial cryo-EM model. As discussed in the Results section, the ensembles generated by simulations started from different conformations overlap in conformational space
**2. Connection to experiments**			
2a. Are calculations provided that can connect to experiments (*e.g.* loss or gain in function from mutagenesis, binding assays, NMR chemical shifts, J-couplings, SAXS curves, interaction distances or FRET distances, structure factors, diffusion coefficients, bulk modulus and other mechanical properties, *etc.*)?	☒		Fig. 3b, 3c. Correlation coefficient of maps generated from MD ensembles compared to the cryo-EM map.
**3. Method choice**			
3a. Is it described in the text what force field and water model are used and why?	☒		This information is contained in the Methods section: Molecular dynamics (MD) simulations.
3b. Do simulations contain membranes, membrane proteins, intrinsically disordered proteins, glycans, nucleic acids, polymers, or cryptic ligand binding?	☒	☐	Response not needed if **N/A**
If 3b is **YES**, are enhanced sampling methods used?	☐	☒	Response not needed if **N/A**
If enhanced sampling methods are used, are the convergence criteria clearly stated?	☐		

If 3b is **YES**, is it explained in the text why or why not enhanced sampling methods are used?	☒		Since the nucleotides are a part of the very rigid ribosomal RNA as evident from the text, they do not require enhanced sampling.
**4. Code and reproducibility**			
4a. Is a table provided describing the system setup, such as simulation box dimensions, total number of atoms, total number of water molecules, salt concentration, lipid composition (number of molecules and type)?	☒		All the information is provided in the Methods section: Molecular dynamics (MD) simulations and in the repository: https://doi.org/10.5281/zenodo.10874716
4b. Is it described in the text what simulation and analysis software and which versions are used?	☒		The simulation and analysis software are described in the Methods section: Molecular dynamics (MD) simulations.
4c. Are initial coordinate and simulation input files and a coordinate file of the final output provided as supplementary files or in a public repository?	☒		These files are publicly available on zenodo.org: https://doi.org/10.5281/zenodo.10874716
4d. Is there custom code or custom force field parameters?	☐	☒	Response not needed if **N/A**
If **YES**, are they provided as supplementary profiles or in a public repository?	☐		
