# Supplementary material for: Multimodal binding and inhibition of bacterial ribosomes by the antimicrobial peptides Api137 and Api88
Source: Nat Commun. 2024 May 10;15:3945. doi: 10.1038/s41467-024-48027-4 (PMC11087509; doi:10.1038/s41467-024-48027-4)
Supplement: Supplementary file 3 — Reporting Summary [file 41467_2024_48027_MOESM3_ESM.pdf]

nature portfolio

Corresponding author(s): Rainer Nikolay, Christian Spahn, Ralf Hoffmann

Last updated by author(s): 26/03/24

Reporting Summary

Nature Portfolio wishes to improve the reproducibility of the work that we publish. This form provides structure for consistency and transparency in reporting. For further information on Nature Portfolio policies, see our Editorial Policies and the Editorial Policy Checklist.

Statistics

For all statistical analyses, confirm that the following items are present in the figure legend, table legend, main text, or Methods section.

n/a	Confirmed	
☐	☒	The exact sample size (*n*) for each experimental group/condition, given as a discrete number and unit of measurement
☐	☒	A statement on whether measurements were taken from distinct samples or whether the same sample was measured repeatedly
☐	☒	The statistical test(s) used AND whether they are one- or two-sided<br>*Only common tests should be described solely by name; describe more complex techniques in the Methods section.*
☒	☐	A description of all covariates tested
☒	☐	A description of any assumptions or corrections, such as tests of normality and adjustment for multiple comparisons
☐	☒	A full description of the statistical parameters including central tendency (e.g. means) or other basic estimates (e.g. regression coefficient) AND variation (e.g. standard deviation) or associated estimates of uncertainty (e.g. confidence intervals)
☐	☒	For null hypothesis testing, the test statistic (e.g. *F*, *t*, *r*) with confidence intervals, effect sizes, degrees of freedom and *P* value noted<br>*Give P values as exact values whenever suitable.*
☒	☐	For Bayesian analysis, information on the choice of priors and Markov chain Monte Carlo settings
☒	☐	For hierarchical and complex designs, identification of the appropriate level for tests and full reporting of outcomes
☒	☐	Estimates of effect sizes (e.g. Cohen's *d*, Pearson's *r*), indicating how they were calculated

Our web collection on statistics for biologists contains articles on many of the points above.

Software and code

Policy information about availability of computer code

Data collection	Cryo-EM data were collected using the EPU software version 2.8 (FEI, Netherlands) using AutoCTF function of Sherpa (version 2.11.1). No software was used.
Data analysis	GraphPad Prism10 v10.1.2 for in vitro translation and statistics, Microsoft Excel and SigmaPlot13 for binding curves. EPU 2.8.1 (Thermo Fischer Scientific) was used for cryo-EM data acquisition. WARP was used for pre-processing micrographs, correcting CTFs, picking and extracting particles. CryoSPARC and Relion 3. were used for initial reconstructions, 3D classifications, final refinements and to calculate global and local resolution. Coot v0.9.6 was used for manual model building. Phenix 1.20 was used for model refinement , validation and statistics. Figures were generated using ChimeraX v1.5, Adobe Illustrator and Microsoft PowerPoint. MD simulations were prepared, performed and analyzed using GROMACS 2023 with the implemented LINCS, ANAEIG, COVAR and GEN IONversions. For preparation WHATIF 20071220-093 was used. Extreme states of MD simulations were visualized using PyMOL. 2D histograms were plotted using Python using matplotlib and Seaborn. Gromaps was used to calculate density maps from MD structures and compared to experimentally extracted maps using ChimeraX.

For manuscripts utilizing custom algorithms or software that are central to the research but not yet described in published literature, software must be made available to editors and reviewers. We strongly encourage code deposition in a community repository (e.g. GitHub). See the Nature Portfolio guidelines for submitting code & software for further information.

Data

Policy information about availability of data

All manuscripts must include a data availability statement. This statement should provide the following information, where applicable:

- Accession codes, unique identifiers, or web links for publicly available datasets
- A description of any restrictions on data availability
- For clinical datasets or third party data, please ensure that the statement adheres to our policy

Cryo-EM density maps and atomic models are stored in EMDB and PDB as follows:
EMD-19426 [https://www.ebi.ac.uk/emdb/EMD-19426],
8RPY [https://doi.org/10.2210/pdb8RPY/pdb] (50S in complex with Api137);
EMD-19427 [https://www.ebi.ac.uk/emdb/EMD-19427],
8RPZ [https://doi.org/10.2210/pdb8RPZ/pdb] (50S in complex with Api88 conf. I);
EMD-19428 [https://www.ebi.ac.uk/emdb/EMD-19428],
8RQ0 [https://doi.org/10.2210/pdb8RQ0/pdb] (50S in complex with Api88 conf. II);
EMD-19429 [https://www.ebi.ac.uk/emdb/EMD-19429],
8RQ2 [https://doi.org/10.2210/pdb8RQ2/pdb] (50S in complex with Api88 conf. III).
Mass spectrometry proteomics data have been deposited to panorama with the ProteomeXchange ID PXD044892 [https://doi.org/10.6069/3f7w-2t74]. Molecular dynamics simulation data are publicly available on zenodo.org: [https://doi.org/10.5281/zenodo.10874716]. All additional data needed to evaluate the conclusions in the paper are present in the paper and/or the Supplementary Materials. Source data are provided with this paper.

Research involving human participants, their data, or biological material

Policy information about studies with human participants or human data. See also policy information about sex, gender (identity/presentation), and sexual orientation and race, ethnicity and racism.

Reporting on sex and gender	N/A
Reporting on race, ethnicity, or other socially relevant groupings	N/A
Population characteristics	N/A
Recruitment	N/A
Ethics oversight	N/A

Note that full information on the approval of the study protocol must also be provided in the manuscript.

Field-specific reporting

Please select the one below that is the best fit for your research. If you are not sure, read the appropriate sections before making your selection.

☒ Life sciences ☐ Behavioural & social sciences ☐ Ecological, evolutionary & environmental sciences

For a reference copy of the document with all sections, see nature.com/documents/nr-reporting-summary-flat.pdf

Life sciences study design

All studies must disclose on these points even when the disclosure is negative.

Sample size	Sample sizes and number of replicates were based on successful experiments from previous publications. Cryo-EM data were collected for a sufficient duration to generate cryo-EM maps with a resolution below 3 angstroms.
Data exclusions	Micrographs with low estimated resolution or poorly fitted CTFs were discarded, along with particles that clustered into poorly defined classes during 3D classification.
Replication	In vitro translation assay was performed in three independent experiments. Kd values were determined in one experiment as triplicates. Ki values were determined in two independent experiments as duplicates. Figure legends state how often experiments have been reproduced. All experiments were successful and produced converging results.
Randomization	Not relevant, as characterized compounds were individually tested and result independent of the investigator`s prior knowledge or hypothesis.
Blinding	No relevant, as the results are independent of the investigator`s prior knowledge or hypothesis.

Reporting for specific materials, systems and methods

We require information from authors about some types of materials, experimental systems and methods used in many studies. Here, indicate whether each material, system or method listed is relevant to your study. If you are not sure if a list item applies to your research, read the appropriate section before selecting a response.

Materials & experimental systems

n/a	Involved in the study
☐	☒ Antibodies
☒	☐ Eukaryotic cell lines
☒	☐ Palaeontology and archaeology
☒	☐ Animals and other organisms
☒	☐ Clinical data
☒	☐ Dual use research of concern
☒	☐ Plants

Methods

n/a	Involved in the study
☒	☐ ChIP-seq
☒	☐ Flow cytometry
☒	☐ MRI-based neuroimaging

Antibodies

Antibodies used

mouse IgG2a anti-Biotin monoclonal antibody clone 4C7.D5 (# 200-301-098; https://www.rockland.com/categories/primary-antibodies/biotin-monoclonal-antibody-200-301-098/) ordered by ThermoFisher (Life Technologie; # 200301098; https://www.thermofisher.com/antibody/product/Biotin-Antibody-clone-4C7-D5-Monoclonal/200-301-098; Lot XC3543791). A goat IgG anti-mouse IgG-Fc conjugated to gold nanoparticles provided in the PIA-Pink-Mouse kit (PiNa-Tec, Hamburg, Germany; # 104-20; lot 23030602-2; http://pina-tec.de/?page_id=265).

Validation

Antibodies were obtained as part of a validated kit.
